# PCID2 Subunit of the *Drosophila* TREX-2 Complex Has Two RNA-Binding Regions

**DOI:** 10.3390/cimb45070357

**Published:** 2023-07-04

**Authors:** Yulia A. Vdovina, Maria M. Kurshakova, Sofia G. Georgieva, Daria V. Kopytova

**Affiliations:** Engelhardt Institute of Molecular Biology, Russian Academy of Sciences, 119991 Moscow, Russia

**Keywords:** TREX-2, EMSA, PCID2

## Abstract

*Drosophila* PCID2 is a subunit of the TREX-2 mRNA nuclear export complex. Although the complex has long been studied in eukaryotes, it is still unclear how TREX-2 interacts with mRNA in multicellular organisms. Here, the interaction between *Drosophila* PCID2 and the *ras2* RNA was studied by EMSA. We show that the C-terminal region of the WH domain of PCID2 specifically binds the 3′-noncoding region of the *ras2* RNA. While the same region of PCID2 interacts with the Xmas-2 subunit of the TREX-2 complex, PCID2 interacts with RNA independently of Xmas-2. An additional RNA-binding region (M region) was identified in the N-terminal part of the PCI domain and found to bind RNA nonspecifically. Point mutations of evolutionarily conserved amino acid residues in this region completely abolish the PCID2–RNA interaction, while a deletion of the C-terminal domain only partly decreases it. Thus, the specific interaction of PCID2 with RNA requires nonspecific PCID2–RNA binding.

## 1. Introduction

The *Drosophila* PCID2 protein is a component of the TREX-2 complex, which is evolutionarily conserved in eukaryotes and is responsible for mRNA nuclear export [1,2,3,4,5]. TREX-2 has been intensely studied in yeast, *Drosophila*, and humans [4,5,6,7,8]. Apart from playing a role in mRNA export, TREX-2 is involved in positioning genes in the nucleus, preventing genome instability, and regulating gene transcription [4,8,9,10,11,12]. A knockdown of TREX-2 subunits distorts mRNA export and leads to mRNA accumulation in the cell nucleus [1,3,4,5,9,13,14,15]. The TREX-2 complex occurs in the nucleoplasm and at the periphery of the nucleus, colocalizing with the nuclear pore complex (NPC) [4,9,16,17]. TREX-2 proteins have been found to interact with the cargo mRNA, components of the mRNP particle, mRNA nuclear export receptors, and NPC proteins during mRNA export [4,5,9,14,18,19,20,21]. TREX-2 interacts with the Mediator complex and regulates the assembly of Mediator with Cdk8 kinase [22]. TREX-2 additionally interacts with the SAGA transcriptional complexes, and the interaction determines the physical attachment of transcriptionally active loci to the nuclear pore [4,9,23].

*Drosophila* Xmas-2/yeast Sac3 is a scaffold TREX-2 subunit, around which dPCID2/yThp1 and dENY2/ySus1 are organized [7,16,24,25]. Additionally, it has been found that Sem1, a small, negatively charged protein, is associated with TREX-2 and stabilizes the complex in yeast [7,26]. PCID2 is a subunit responsible for the TREX-2–mRNA interaction [7]. Its major part is the PCI domain, which is a bipartite fold that consists of a structurally conserved C-terminal winged-helix (WH) domain and a more divergent N-terminal HD domain and is based on a stack of α-helices [7,25]. The WH domain is a common nucleic acid-binding motif shown to interact with dsDNA, ssDNA, and RNA [7,26,27,28].

Crystallization of yeast Thp1-Sem1 and Sac3 has shown that Sem1 is associated with the HD domain of Thp1, while Thp1 is associated with Sac3 through its C-terminal WH domain [7]. The WH domains of Sac3 and Thp1 form a common, positively charged surface, which mediates the interaction of the complex with nucleic acids [7]. In EMSA experiments, the individual components of the complex (Thp1-Sem1 and Sac3 250–563) do not interact with RNA, but efficiently bind RNA after being preincubated together [7]. However, an earlier study has demonstrated that Thp1 is capable of nucleic acid binding *in vitro* regardless of the other proteins of the complex [29]. While it has been demonstrated that human PCID2 is associated with Sem1 in a way similar to that of their yeast homologues [7], the interactions of *Drosophila* PCID2 and the whole *Drosophila* TREX-2 complex with mRNA has not been studied yet.

In particular, it is unclear whether TREX-2 binds a specific region of mRNA and which regions of PCID2 are responsible for the interaction with RNA. Another unknown issue is whether the TREX-2 subunits dPCID2 and dXmas-2 interact with RNA independently of each other.

The interaction of *Drosophila* PCID2 with mRNA was studied in this work. We showed that a C-terminal fragment present in a WD domain-homologous RNA-interacting fragment of yeast Thp1 specifically interacts with the 3′-noncoding region of the *ras64B* (*ras2*) mRNA. The fragment is also necessary for the interaction with Xmas-2, but Xmas-2 is not required for the PCID2 interaction with the *ras2* mRNA in vitro. An additional RNA-binding region, which interacts with RNA nonspecifically, is in the N-terminal part of the PCI domain. Point mutations of evolutionarily conserved amino acids in this region totally abolish the PCID2–RNA interaction. Thus, PCID2 binds with RNA independently of the other TREX-2 subunits via its two domains, and a distorted interaction with RNA in the N-terminal part of the PCI domain disturbs the RNA binding with the C-terminal domain of the protein.

## 2. Materials and Methods

Sequences coding for the full-size PCID2 and its truncated and mutated variants were cloned into pAc5.1/V5-His (Invitrogen, Waltham, MA, USA) (C-terminally tagged HA epitope) and pGEX-5X-1 (GE Healthcare, Chicago, IL, USA) (N-terminally tagged GST epitope). Sequences coding for the N (residues 1–135), M (136–282), and C (283–395) domains of PCID2 were cloned into pET28a (Novagene, Beijing, China) (C-terminally tagged His epitope). A sequence coding for the full-size Xmas-2 N-terminally fused with three FLAG epitopes was cloned into the pAc5.1/V5-His vector (Invitrogen).

The recombinant GST- or His-tagged proteins were expressed in *E. coli* BL21 cells at 20 °C for 24 h. Cells were collected by centrifugation and stored at −70 °C. The recombinant proteins with the GST-epitope tag were purified from cell lysates by binding to Glutathione SepharoseTM 4 fast flow (Cytiva, Marlborough, MA, USA) and subsequently eluted by displacement with glutathione according to the manufacturer’s recommendations (GE Healthcare). The recombinant proteins with the His-epitope tag were purified from cell lysates by binding to Ni-SepharoseTM (GE) and subsequently eluted by increasing the imidazole concentration according to the manufacturer’s recommendations (GE Healthcare).

### 2.1. Synthesis of Radiolabeled RNA Fragments

DNA templates for [α-32P] RNA synthesis of the *ras2* mRNA fragments were prepared from pBluescript SK (–)-*ras2* plasmids by linearizing the cloned sequences from the 3′ end for the sense fragments. Plasmids were engineered to contain the DNA sequences corresponding to the following *ras2* mRNA fragments: *ras2* fr1 (nucleotides 1–332), *ras2* fr2 (333–656), *ras2* fr3 (657–914), *ras2* fr4 (915–1326), *ras2* fr5 (1327–1597), *ras2* fr4_1 (915–1017), *ras2* fr4_2 (1018–1222), and *ras2* fr4_3 (1223–1326). Radiolabeled RNAs were prepared using the RNA Labeling Mix and T7 and T3 polymerases (Roche Diagnostics, Rotkreuz, Switzerland), treated with RNase-free DNase I, and purified using a RNeasy kit (Qiagen, Venlo, The Netherlands). Each radiolabeled RNA was analyzed by agarose gel electrophoresis and quantified by UV spectrometry.

### 2.2. Electrophoretic Mobility Shift Assay (EMSA)

Purified recombinant proteins corresponding to the full-size PCID2; truncated PCID2; mutated PCID2 variants; and the N, M, and C parts of PCID2 were incubated with the radiolabeled RNA fragments in 20 μL of a binding buffer, which contained 25 mM Tris-HCl, pH 8.0, 100 mM NaCl, 0.5% Triton X-100, 5% glycerol, 1 mM EDTA, 1 mM DTT, 25x PIC, and 40x RiboLock (Thermo Scientific, Waltham, MA, USA), at 4 °C for 1 h. After binding was completed, a PAGE loading buffer was added to the samples, and the samples were applied to a native 5% polyacrylamide gel. Electrophoretic separation was carried out in a 0.5x TBE buffer at 150 V at 4 °C for 80 min. The radioactive signal was detected using a Cyclone Storage Phosphor Screen device. The signal from each lane (area) was quantitated using the ImageJ program. Direct titration reactions were plotted as bound RNA fraction versus protein concentration, and K_D_ was determined according to [30,31].

### 2.3. Drosophila Cell Culture Extracts

*Drosophila* S2 cells were maintained in Schneider’s insect medium (MilliporeSigma, Burlington, MA, USA) containing 10% fetal bovine serum (HyClone, Logan, UT, USA) at 25 °C. Transient transfection of S2 cells was performed using the Macsfectin™ reagent according to the manufacturer’s recommendations (Miltenyi Biotec, Auburn, CA, USA).

To extract proteins, S2 cells were lysed. Cells were centrifuged at 2000 rpm at 4 °C for 5 min and resuspended in 1 mL of 1x PBS with 25x PIC (Protease Inhibitor Cocktail, Roche, Rotkreuz, Switzerland). The cells were then centrifuged once more at 2000 rpm at 4 °C for 5 min and resuspended in an LB buffer, which contained 10 mM Hepes, pH 7.0, 0.4 M NaCl, 5 mM MgCl2, 0.5% NP-40, 25x PIC, 1 mM DTT, and 0.3 μL of DNAse I, in a proportion of 1:10. The cells were incubated with the LB buffer on ice for 20 min, and the lysate was further centrifuged at 13,000 rpm at 4 °C for 15 min.

Immunoprecipitation was performed as described [32], using extracts pretreated with DNase I (Thermo Scientific, Waltham, MA, USA, 0.6 U/mL), RNase (Thermo Scientific, 10 U/mL), and antibodies against the FLAG and HA epitopes.

### 2.4. Antibodies

Antibodies against PCID2 [14], the FLAG epitope (Cell Signaling Technology, Danvers, MA, USA), and the HA epitope (MilliporeSigma, Burlington, MA, USA) were used.

### 2.5. Statistical Analysis

Error bars represent SD from at least three independent experiments. All data are presented as mean ± SD, and Student’s *t*-test was used to compare the control and treatment groups. An asterisk (*) indicates statistical significance with *p*-value < 0.05; (**) indicates statistical significance with *p*-value < 0.01.

### 2.6. Biomolecular Resources

The predicted *Drosophila* PCID2 structure (AF ID: AF-Q9VTL1-F1) was provided by the AlphaFold Protein Structure Database (ebi.ac.uk, accessed on 28 October 2022), which has been created in partnership with EMBL’s European Bioinformatics Institute (EMBL-EBI) [33,34]. The PCID2 structure was visualized using UCSF ChimeraX software, which has been developed by the Resource for Biocomputing, Visualization, and Informatics at the University of California, San Francisco, with support from National Institutes of Health R01-GM129325 and the Office of Cyber Infrastructure and Computational Biology, National Institute of Allergy and Infectious Diseases [35]. The electrostatic properties of the protein surface were computed using the APBS web service (poissonboltzmann.org, accessed on 23 April 2023) [36].

## 3. Results

### 3.1. PCID2 Specifically Interacts with a Fragment of the 3′-Noncoding Region of the ras2 mRNA Independently of Xmas-2

**Figure 1:**
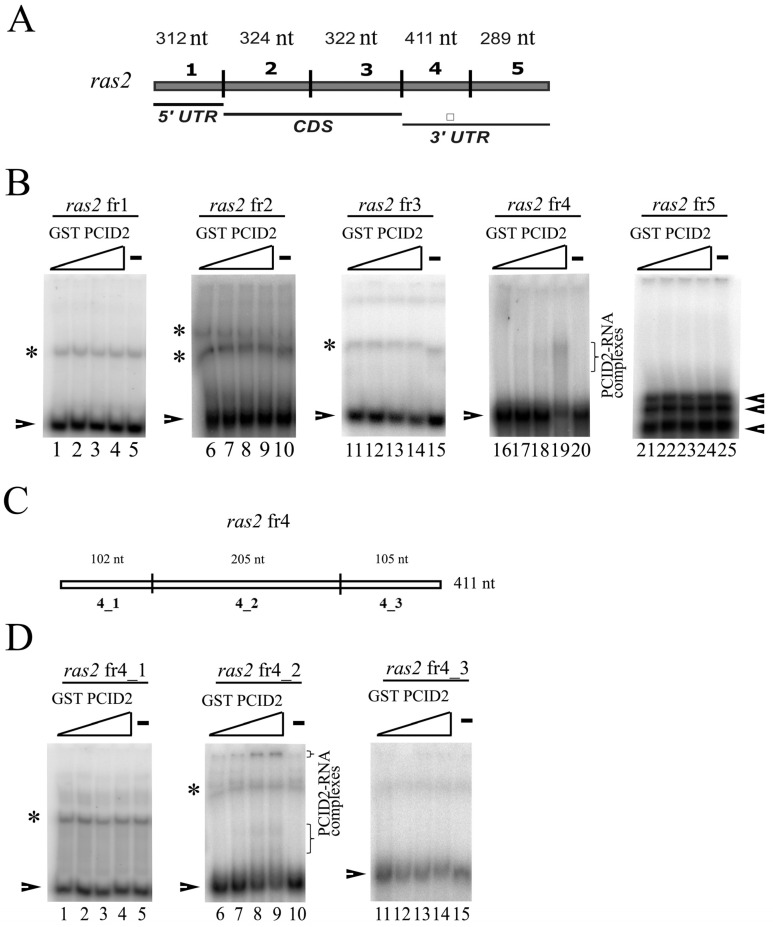
EMSA analysis of the *Drosophila* PCID2 interactions with the *ras2* RNA fragments. (**A**) Schematic subdivision of the *ras2* (*ras64B*) mRNA into the fragments used in the RNA binding experiments. Indicated are the mRNA regions to which the fragments used in the experiment correspond. (**B**,**D**) Replicas from native 5% polyacrylamide gels are shown. Arrowheads show the migration of major bands and asterisks show the migration of minor bands of free RNAs. The resulting protein–RNA complexes are shown in brackets. The concentration of each RNA fragment in the binding reaction was 25 nM. The numbering of the RNA fragments is as shown in (**A**,**C**). There was no PCID2–GST protein in the first lane of each replica (**B**: lanes 1, 6, 11, 16, 21; **D**: 1, 6, and 11); the PCID2–GST concentration was 6 nM in the second lane of each replica, 12 nM in the third lane of each replica, and 15 nM in the fourth lane of each replica. GST alone was added to the binding reaction at 125 nM (the fifth lane of each replica). (**C**) Schematic subdivision of the *ras2* fragment 4 into the three smaller fragments used in the RNA binding experiments. Indicated are the mRNA regions to which the fragments used in the experiment correspond. * indicates the significance with *p*-value < 0.05. ** indicates the statistical significance with *p*-value < 0.01.

A direct interaction of *Drosophila* PCID2 with RNA was studied by EMSA. The *ras2* (*ras64B*) mRNA was used in experiments. The choice was explained by our previous finding that Xmas-2 and PCID2 bound the *ras2* mRNA in RNA immunoprecipitation experiments and the RNA pull-down assay [14,19]. The *ras2* mRNA sequence was divided into five approximately equal fragments (Figure 1A). *ras2* fr1 corresponded to the 5′-noncoding region, *ras2* fr2 and *ras2* fr3 to the coding region, and *ras2* fr4 and *ras2* fr5 corresponded to the 3′-noncoding region of the *ras2* mRNA. The respective radiolabeled RNA fragments were synthesized using T3 and T7 RNA polymerases and [α-32P]UTP. A sequence encoding the full-size PCID2 was cloned in pGEX 5.1, and the protein was expressed in a bacterial system. Each RNA fragment was tested for binding with the full-size PCID2 fused with the GST epitope. *ras2* fr4, which corresponded to the 3′-noncoding region of the *ras2* mRNA, was found to interact with PCID2 in EMSA experiments (Figure 1B).

**Figure 2:**
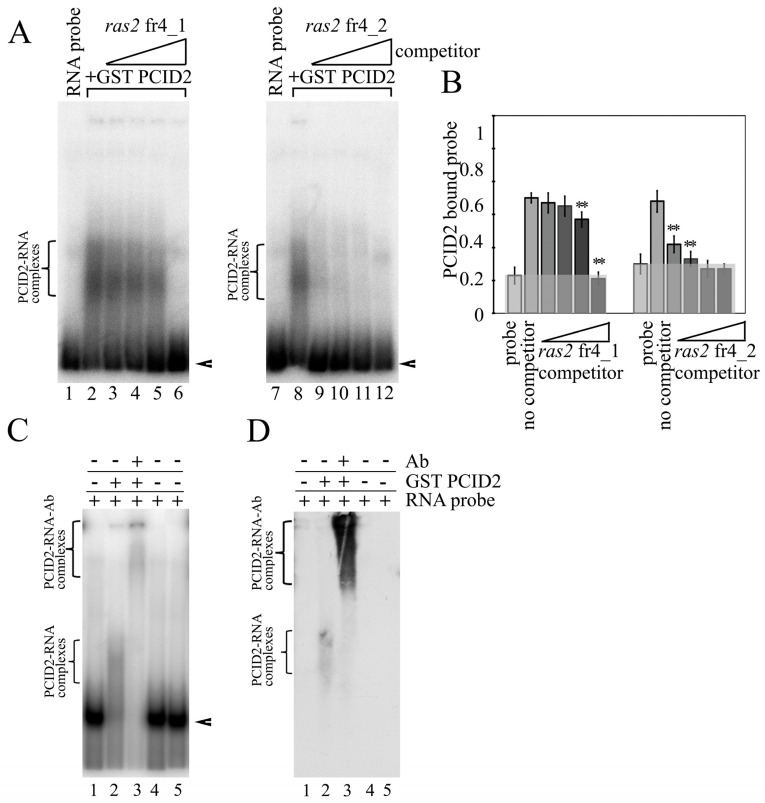
PCID2 specifically binds to *ras2* mRNA fragment 4_2. (**A**) A gel-shifted complex formed of PCID2-GST and *ras2* mRNA fragment 4_2 in the presence of cold fragment 4_1 (left panel) or 4_2 (right panel). Arrowheads show the migration of free RNAs. An arrow indicates the major high-order gel-shifted complex. The concentration of PCID2-GST was 50 nM. [^32^P] UTP-labelled RNA (25 nM) was incubated with competitor RNA fragment 4_1 or 4_2, which was used at increasing molar ratios of 2.5×, 25×, 50×, and 250×. (**B**) Results (mean ± SD) of three independent competition assays of individual RNA competitors were analyzed using phosphoimaging; (**) indicates the statistical significance with *p*-value < 0.01. (**C**) The RNA-protein complex PCID2-fragment 4_2 could be supershifted by antibodies to PCID2. The concentration of the 4_2 RNA fragment was 25 nM, PCID2-GST or GST (control) was used at 50 nM. The antibodies (1.5 µL) were added to the reaction, and the mixture was further incubated for 30 min. The complexes were analyzed by native 5% PAGE. Arrowheads show the migration of free RNA. Brackets indicate the major high-order gel-shifted complexes. (**D**) Western blot of (**C**); the blot was stained with the antibodies to PCID2.

To determine the binding site in the *ras2* mRNA more precisely, *ras2* fr4 was divided into three parts (Figure 1C). Only the *ras2* 4_2 fragment was found to interact with PCID2 in EMSA experiments (Figure 1D). The specificity of the interaction was confirmed in a competition assay (Figure 2A,B). *ras2* 4_1 (left panel) and *ras2* 4_2 (right panel) fragments were used as cold competitors. A competitor was added to the reaction in 0.5- to 250-fold excess over the hot 4_2 fragment in a gradient manner. Figure 2B shows the level of binding of GST-PCID2 with hot *ras2* fragment 4_2. The interaction of the hot 4_2 fragment with PCID2 decreased gradually with a minimal increment in the presence of cold fragment 4_1, while cold fragment 4_2 competed with its hot counterpart and already decreased its interaction with the protein when used in a minor excess (Figure 2B).

To better visualize the specific interaction of PCID2 with fragment 4_2 of the *ras2* mRNA, supershift EMSA was carried out with anti-PCID2 antibodies (Figure 2C). The experiment showed that a supershift, a larger antibody–protein–RNA complex (Figure 2C, lane 3), formed in addition to the protein–RNA complex (Figure 2C, lane 2) when antibodies against PCID2 were added. Western blotting was performed to verify the result (Figure 2D). Membrane hybridization with the anti-PCID2 antibodies also detected the formation of the antibody–protein–RNA complex (Figure 2D, lane 3). To obtain quantitative information on PCID2 binding specificity, we determined the apparent K_D_ of RNA fragment 4_2. Apparent K_D_ values of about 37 nM were observed for *ras2* fragment 4_2, indicating high-affinity binding to PCID2 (Figure S1).

Thus, PCID2 directly binds RNA and specifically interacts with a sequence located in the 3′-noncoding region of the *ras2* mRNA. The experiments demonstrated additionally that PCID2 interacts with RNA without being associated with other TREX-2 subunits, such as Xmas-2, which has been implicated in the binding in yeast [7].

### 3.2. The C-Terminal Fragment of the PCI domain Interacts with the 3′-Noncoding Region of the ras2 mRNA

PCID2 and its homologs are structurally similar; their evolutionarily conserved C-terminal region (the PCI domain) includes the highly structurally conserved WH domain [7,26]. The C-terminal part of the WH domain of the yeast PCID2 homolog has been shown to interact with the yeast Xmas-2 homolog to form a platform for interactions with an RNA molecule. Certain amino acid sequences were identified as responsible for the interaction of the protein with RNA within the TREX-2 complex [7].

**Figure 3:**
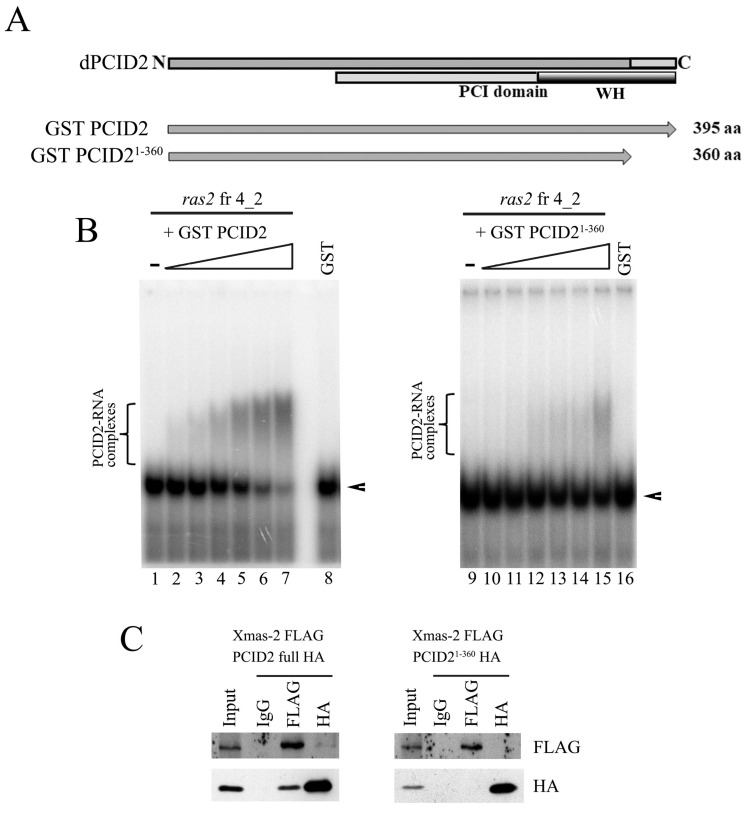
The C-terminal domain of PCID2 is involved in interacting with the 3′-noncoding region of the *ras2* mRNA and Xmas-2. (**A**) The domain structure of PCID2. The PCI domain and the WH domain responsible for interactions with RNA and Xmas-2 (by homology with yeast proteins) are shown. The full-size and truncated PCID2 proteins taken in the experiment are indicated. (**B**) The formation of gel-shifted complexes of GST-PCID2 (left panel) and GST-PCID2^1-360^ (right panel) and *ras2* fragment 4_2. [^32^P] UTP-labelled, in vitro-transcribed fragment 4_2 of the *ras2* RNA (25 nM) was incubated with increasing amounts (0, 5, 12.5, 25, 50, 87, and 125 nM) of purified GST or the GST–PCID2 or GST–PCID2^1−360^ fusion protein. GST alone was added to the binding reaction at 125 nM. The complexes were analyzed by native 5% PAGE. Arrowheads show the migration of free RNAs. Brackets indicate the major high-order gel-shifted complexes. (**C**) The C-terminal domain of PCID2 interacts with Xmas-2. HA-tagged full-size Xmas-2 was co-expressed with FLAG-tagged PCID2 or PCID2^1−360^ in S2 cells. Their interaction was tested in coimmunoprecipitation experiments with antibodies against the FLAG or HA epitope. PCID2^1−360^ does not interact with Xmas-2.

We studied the role that the homologous C-terminal region of PCID2 plays in its interaction with RNA. We constructed a PCID2 devoid of the 35 C-terminal amino acid residues homologous to those shown to be involved in interactions in yeast (PCID2^1–360^). The full-size PCID2 and PCID2^1–360^, each fused with the GST epitope, were tested for binding fragment 4_2 of the *ras2* mRNA by EMSA (Figure 3A). PCID2^1–360^ far less efficiently interacted with the RNA fragment as compared with the full-size PCID2 (Figure 3B), indicating that the C-terminal domain plays an important role in the interaction with RNA. However, it should be noted that the interaction with RNA was not completely abolished. The finding indicates that another PCID2 region is probably also involved in interacting with the *ras2* mRNA.

### 3.3. The C-Terminal Fragment of the PCI domain Interacts with the 3′-Noncoding Region of the ras2 mRNA

A role in interacting with Xmas-2 was additionally demonstrated for the C-terminal domain of PCID2 in our experiments. The interaction of the truncated PCID2 (PCID2^1–360^) with Xmas-2 was studied in *D. melanogaster* S2 cells. PCID2^1–360^ was fused with the HA epitope, Xmas-2 was fused with the FLAG epitope, and the two proteins were coexpressed in S2 cells. The results of protein coimmunoprecipitation from a cell lysate are shown in Figure 3C. PCID2^1–360^ did not interact with Xmas-2 in the experiment (Figure 3C, right panel). At the same time, the full-size PCID2 and Xmas-2 interacted with each other (Figure 3C, left panel). Thus, the domain utilized by *D. melanogaster* PCID2 to interact with Xmas-2 is the same that the domain utilized by its yeast homolog [7]. Still, the PCID2 interaction with the specific RNA fragment was shown to be independent of Xmas-2 in our experiments.

### 3.4. There Is an Additional RNA Interaction Region in PCID2

**Figure 4:**
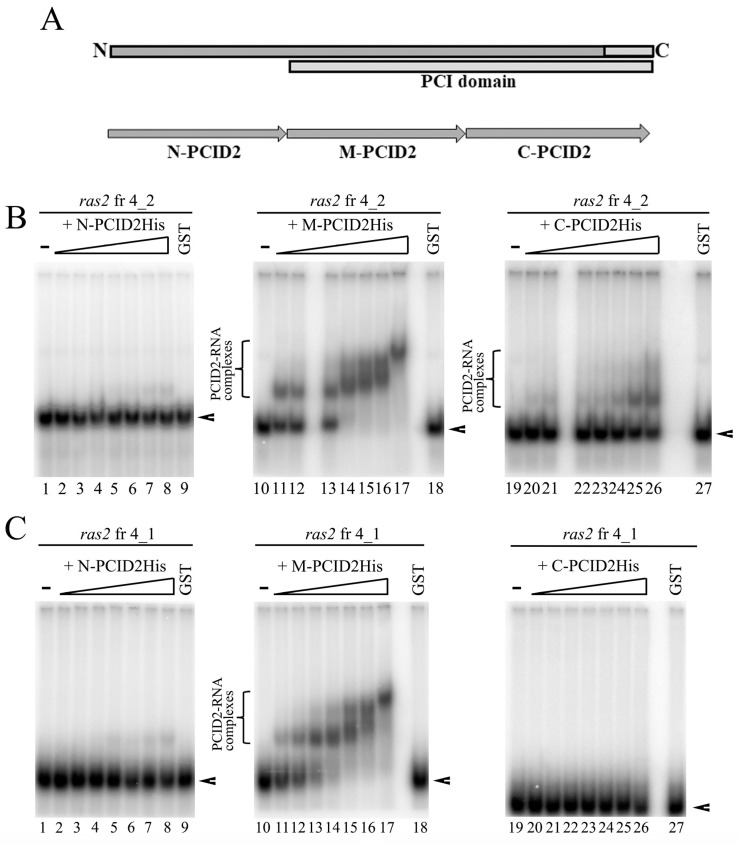
The C domain of PCID2 specifically binds with *ras2* mRNA fragment 4_2, while its M domain binds nonspecifically. (**A**) Schematic subdivision of PCID2 into N, M, and C fragments. The proteins fused with the His tag were expressed in a bacterial system, purified on Ni–NTA resin, and used in the binding reaction. (**B**) [^32^P] UTP-labelled, in vitro-transcribed *ras2* mRNA fragment 4_2 (25 nM) was incubated with increasing amounts (0, 2.5, 5, 10, 15, 20, 25, and 50 nM) of purified N-PCID2-His (left panel), M-PCID2-His (middle panel), or C-PCID2-His (right panel). GST alone was added to the binding reaction at 125 nM. The complexes were analyzed by native 5% PAGE. Brackets indicate the major high-order gel-shifted complexes. Arrowheads show the migration of free RNAs. (**C**) [^32^P] UTP-labelled, in vitro-transcribed *ras2* mRNA fragment 4_1 (25 nM) was incubated with increasing amounts (0, 2.5, 5, 10, 15, 20, 25, and 50 nM) of purified N-PCID2-His (left panel), M-PCID2-His (middle panel), or C-PCID2-His (right panel). GST alone was added to the binding reaction at 125 nM. The complexes were analyzed by native 5% PAGE. A bracket indicates the major high-order gel-shifted complexes. Arrowheads show the migration of free RNAs.

Because the deletion of the C-terminal domain of PCID2 did not fully abolish its interaction with RNA, additional RNA-binding sites were assumed for PCID2. To identify the respective regions, we engineered the N-PCID2, M-PCID2, and C-PCID2 constructs, in which the His epitope was fused with the approximately equal (about 130 amino acids) N-terminal, middle, and C-terminal fragments of PCID2, respectively. A fragmentation scheme is shown in Figure 4A. N-PCID2 corresponded to the N-terminal PCID2 part, which does not contain known domains. M-PCID2 included the N part of the PCI domain, while C-PCID2 corresponded to the C-terminal part of PCI domain, including the WH domain, which contains an RNA-binding motif. The PCID2 fragments were tested for interactions with *ras2* RNA fragment 4_2 by EMSA (Figure 4B). The C-PCID2 was found to interact with *ras2* fragment 4_2 (K_D_~60 nM), which is in line with the results shown above (Figure 3B). N-PCID2 did not virtually bind with RNA. We observed the strong interaction with the *ras2* RNA for the M-PCID2 fragment (K_D_~11 nM). M-PCID2 formed several contacts with fragment 4_2 as could be seen from the migration pattern of the RNA–protein complex in gel. Thus, the N-terminal part of the PCI domain harbors an additional region responsible for RNA binding.

Then, we checked whether the interaction of M-PCID2 with RNA is specific. The PCID2 regions were tested for interactions with *ras2* RNA fragment 4_1, which does not interact with the full-size PCID2, as reported above. N-PCID2 and C-PCID2 were not found to interact with fragment 4_1 (Figure 4C). However, M-PCID2 bound the fragment with a considerable efficiency, with K_D_ of about 9.2 nM (Figure 4C). The M region of PCID2 (M-PCID2) was therefore concluded to nonspecifically bind with RNA (Figure 4C).

### 3.5. Point Mutations of the M Domain Abolish PCID2 Binding with RNA

**Figure 5:**
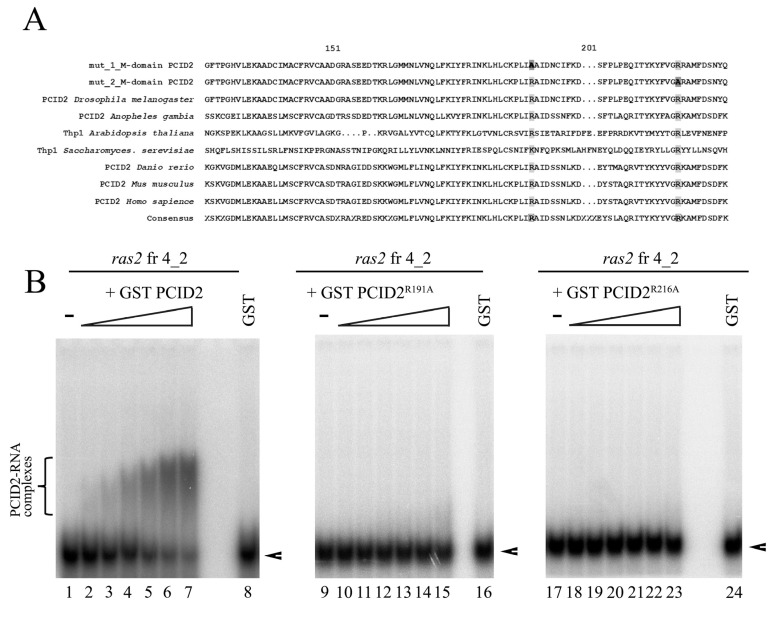
Point mutations of the M domain of PCID2 lead to loss of RNA binding function of PCID2. (**A**) Evolutionary alignment of the M domain of PCID2 in several eukaryote species. The amino acid residues replaced in the first and in the second mutant are framed and highlighted dark. (**B**) GST–PCID2, GST–PCID2^R191A^, and GST–PCID2^R216A^ were expressed in a bacterial system, purified on Glutathione Sepharose, and used in the binding reaction. [^32^P]UTP-labelled, in vitro-transcribed *ras2* mRNA fragment 4_2 (25 nM) was incubated with increasing amounts (0, 5, 12.5, 25, 50, 87, and 125 nM) of purified GST–PCID2 (left panel), GST–PCID2^R191A^ (middle panel), or GST–PCID2^R216A^ (right panel). GST alone was added to the binding reaction at 125 nM. The complexes were analyzed by native 5% PAGE. A bracket indicates the major high-order gel-shifted complexes. Arrowheads show the migration of free RNAs.

Then, we studied the role of the M region in the interaction of the full-size PCID2 with RNA. Conserved amino acid residues potentially responsible for the interaction with RNA were identified in the M domain by evolutionary sequence alignment of PCID2 homologs of various organisms. Two conserved residues, Arg191 and Arg216, were of particular interest according to the prediction of RNA binding sites with the online tools PRIdictor [37] and aaRNA [38]. Two PCID2 mutants fused with the GST epitope were constructed (Figure 5A): Ala was substituted for Arg191 in one of them (PCID2^R191A^) and for Arg216 in the other (PCID2^R216A^). PCID2^R191A^ and PCID2^R216A^ did not bind *ras2* RNA fragment 4_2 in contrast to the wild-type PCID2 (Figure 5B). Thus, the mutations of the M domain fully distorted the interaction of the full-size PCID2 with RNA. Moreover, the results indicate that the C-terminal domain does not interact with RNA in the absence of RNA interactions with the M domain. One may assume that RNA binding with the M region of PCID2 is necessary for RNA binding with its C-terminal domain.

## 4. Discussion

In this work, the RNA-binding domains of *Drosophila* PCID2 were characterized. We showed that the association of *D. melanogaster* PCID2 with RNA may occur independently of the other components of the TREX-2 complex. The 34-residue C-terminal fragment of the PCID2 WH domain specifically interacts with the 3′-noncoding region of the *ras2* mRNA. The fragment corresponds to a region of yeast Thp1, which has been shown to interact with Sac3, and together they form a platform for binding with RNA [7]. Our data show that the C-terminal fragment of PCID2 participates in the interaction with Xmas-2, a homolog of Sac3. However, in contrast to yeast Thp1, *Drosophila* PCID2 does not require Xmas-2 to interact with RNA. These findings agree with the published data that the yeast PCID2 homolog Thp1 may interact with RNA directly, without the involvement of other subunits of the complex [29]. The findings are consistent as well with the results of our previous study, which has shown that PCID2 occurs in the cytoplasm of S2 cells, where it is involved in cytoplasmic mRNA transport and is associated with mRNA independently of the other TREX-2 subunits [14].

We localized the *ras2* mRNA nucleotide sequence to which PCID2 binds specifically. An association of *Drosophila* TREX-2 subunits with the *ras2* mRNA has previously been demonstrated by coimmunoprecipitation [14,19]. In this work, we showed the direct binding of PCID2 with the *ras2* RNA and localized the binding sites to 200 nt of the 3′-noncoding region of the *ras2* mRNA. It is possible to assume that a certain secondary structure forms in the region and that the structure may occur in other mRNAs as well. The issue needs further investigation.

Here, we found the additional RNA-interacting region of PCID2. The region is in the N-terminal part of the PCI domain and binds RNA nonspecifically (M region). Based on the EMSA results, a lack of the C-terminal domain partly distorts the mRNA binding, while mutations of the middle part completely abolish the mRNA binding. The finding makes it possible to conclude that the interaction of the M region with RNA is necessary for the specific RNA binding with the C-terminal region. Primary RNA binding can be assumed to occur in the M region (because its binding with RNA is highly efficient) and to allow the C-terminal domain to specifically interact with the RNA (because its binding with RNA is less efficient). The M region of PCID2 is highly conserved in evolution (up to 91% homology in higher eukaryotes). A similar role is therefore possible to assume for the respective sequences of the PCID2 homologs of other organisms.

**Figure 6:**
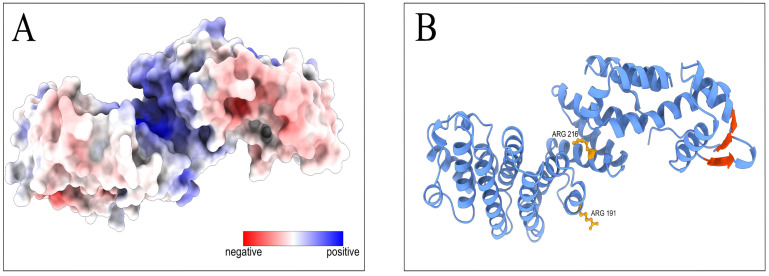
Prediction of RNA-binding domains on the *Drosophila* PCID2 surface. (**A**) The distribution of the electrostatic potential on the predicted PCID2 molecular surface. The color scale of the electrostatic potential is in units of kT/e, ranging from –10 (red) to +10 (blue) at T = 298K. (**B)** The amino acid sites in the M-domain and the β-sheets in the C-domain involved in RNA binding are indicated on the predicted PCID2 structure. The structure was accessed through the AlphaFold Protein Structure Database, which has been created in partnership with EMBL’s European Bioinformatics Institute (EMBL-EBI). Electrostatics analysis was performed using APBS software. Molecular graphics were performed with UCSF ChimeraX, which has been developed by the Resource for Biocomputing, Visualization, and Informatics at the University of California, San Francisco, with support from the National Institutes of Health R01-GM129325 and the Office of Cyber Infrastructure and Computational Biology, National Institute of Allergy and Infectious Diseases.

AlphaFold [33,34] and APBS [36] analyses of the M region structure and properties showed that the M region forms a positively charged groove, a possible surface for RNA-interacting protein domains (Figure 6A). The highly evolutionarily conservative amino acids mutated in our experiments are critical for RNA-binding activity of the positively charged groove (Figure 6B). The confidence of the PCID2 model is represented on Figure S2. The yeast Thp1 domain corresponding to the M region has been shown to interact with the Sem1p protein, and the Thp1–Sem1p complex does not interact with RNA [7]. It can be assumed that the role of Sem1 in the TREX-2 complex is to prevent PCID2 binding with mRNA and thus to regulate the TREX-2–mRNA interaction.

## Data Availability

No new data were created or analyzed in this study. Data sharing is not applicable to this article.

## Supplementary Materials

The following supporting information can be downloaded at: https://www.mdpi.com/article/10.3390/cimb45070357/s1, Figure S1: EMSA analysis of the Drosophila PCID2 interactions with the ras2 fragment 4_2; Figure S2: Confidence of the PCID2 model.
