# Supplementary material for: PCID2 Subunit of the Drosophila TREX-2 Complex Has Two RNA-Binding Regions
Source: Curr Issues Mol Biol. 2023 Jul 4;45(7):5662–76. doi: 10.3390/cimb45070357 (PMC10378293; doi:10.3390/cimb45070357)
Supplement: Supplementary file 1 [file cimb-45-00357-s001.zip › cimb-2454809-supplementary.pdf]

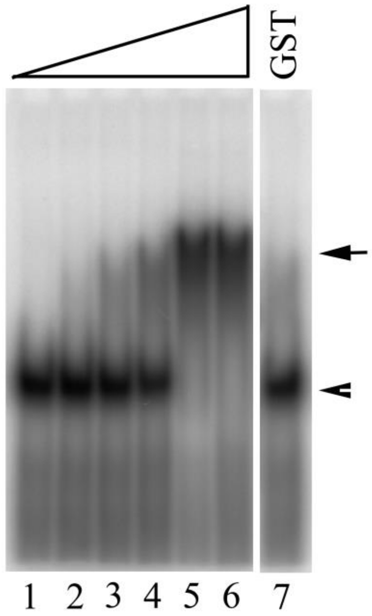

RNA fragments bind to GST-PCID2 with K_D:
ras2 fr4_2 - K_D ~ 50 nM

Figure S1. EMSA analysis of the Drosophila PCID2 interactions with the ras2 fragment 4_2. Replicas from native 5% polyacrylamide gels are shown. An arrow indicates the major high order gel-shifted complex; an arrowhead shows the migration of free RNA. [32P]UTP-labelled RNA fragment 4_2 (25 nM) was incubated with increasing amounts of GST-PCID2 (0, 2.5, 12.5, 25, 125, 250 nM). GST alone was added to the binding reaction at 125 nM (panel 7). KD was determined by counting the radioactive signal of the bound RNA using a Cyclone Storage Phosphor Screen device and calculated using the ImageJ program.

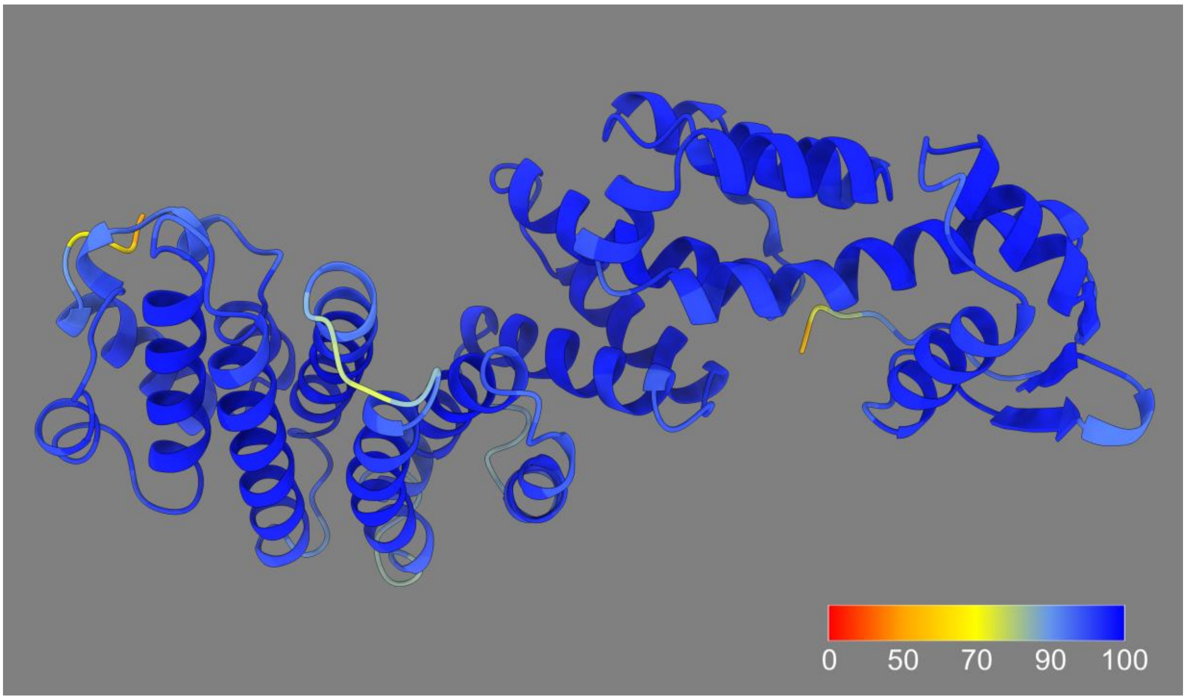

Figure S2. Confidence of the PCID2 model. The scale represents a per-residue confidence score (pLDDT, predicted local distance difference test) in a range of [0, 100]. Blue indicates high confidence predictions (pLDDT > 90), while red indicates very low-confidence predictions (pLDDT < 50). Structural prediction was provided by the AlphaFold Protein Structure Database.
